# Cocoa and Dark Chocolate Polyphenols: From Biology to Clinical Applications

**DOI:** 10.3389/fimmu.2017.00677

**Published:** 2017-06-09

**Authors:** Thea Magrone, Matteo Antonio Russo, Emilio Jirillo

**Affiliations:** ^1^Department of Basic Medical Sciences, Neuroscience and Sensory Organs, University of Bari, Bari, Italy; ^2^MEBIC Consortium, San Raffaele Open University of Rome and IRCCS San Raffaele Pisana of Rome, Rome, Italy; ^3^Fondazione San Raffaele, Ceglie Messapica, Italy

**Keywords:** anti-inflammatory activity, cocoa, dark chocolate, flavanols, nitric oxide, polyphenols, reactive oxygen species, transcription factors

## Abstract

It is well known that cocoa and dark chocolate possess polyphenols as major constituents whose dietary consumption has been associated to beneficial effects. In fact, cocoa and dark chocolate polyphenols exert antioxidant and anti-inflammatory activities switching on some important signaling pathways such as toll-like receptor 4/nuclear factor κB/signal transducer and activator of transcription. In particular, cocoa polyphenols induce release of nitric oxide (NO) through activation of endothelial NO synthase which, in turn, accounts for vasodilation and cardioprotective effects. In the light of the above described properties, a number of clinical trials based on the consumption of cocoa and dark chocolate have been conducted in healthy subjects as well as in different categories of patients, such as those affected by cardiovascular, neurological, intestinal, and metabolic pathologies. Even if data are not always concordant, modifications of biomarkers of disease are frequently associated to improvement of clinical manifestations. Quite interestingly, following cocoa and dark chocolate ingestion, cocoa polyphenols also modulate intestinal microbiota, thus leading to the growth of bacteria that trigger a tolerogenic anti-inflammatory pathway in the host. Finally, many evidences encourage the consumption of cocoa and dark chocolate by aged people for the recovery of the neurovascular unit.

## Introduction

Polyphenols represent a class of natural products that are very spread in the plant kingdom. Mostly, fruits, vegetables, and cereals are considered as major sources of dietary polyphenols, which human beings assume with food. In this context, Mediterranean diet (MED) represents an healthy nutritional regimen based on the consumption of extra virgin olive oil, fruits, vegetables, cereals, legumes, nuts, and seeds plus moderate intake of red wine (1, 2). It has been reported that MED is highly protective against chronic low-grade inflammation, and, in the case of atherosclerosis, stabilizes atheromatous plaques (3). Another study has emphasized the important role played by resveratrol, a non-flavonoid compound contained in red wine, to induce formation of sirtuins (Sirt) which, in turn, exert potent anti-aging effects (4). The MOLI-sani project has documented that in a large prospective cohort study of 24,325 Italian people MED reduced levels of glucose, lipids, C reactive protein (CRP), blood pressure (BP), and 10-year cardiovascular risk (5). Quite interestingly, Morabito and associates (6) have demonstrated that polyphenols contained in fruit juices prevent the post-prandial metabolic stress in humans as well as inflammatory disease outcome.

Taken together, all these are general consideration on dietary polyphenols effects and for more details on their chemical structure and functions, readers are referred to Ref. (7, 8).

With special reference to cocoa, polyphenols are constituents of the beans and their derivatives from the *Theobroma cacao* tree. Cocoa liquor is the paste derived from cocoa beans, the so-called nibs, and it is composed by non-fat cocoa solids and cocoa butter (9). Instead, cocoa powder is obtained by getting rid of some of the cocoa butter from the liquor. Finally, chocolate results from the combination of cocoa liquor with cocoa butter and sugar.

With regard to lipids, cocoa butter contains both monounsaturated and saturated fatty acids (FAs) (10). Oleic acid is the major monounsaturated FA that is present in similar amounts to those contained in the olive oil (10). Conversely, palmitic and stearic acids represent the main saturated FAs. However, stearic acid has been found to be anti-atherogenic, also accounting for one-third of the lipids contained in cocoa butter (11).

Fibers are present in cocoa beans, and their consumption has been shown to improve the low density lipoprotein (LDL):high density lipoprotein (HDL) ratio (12), also reducing risk of type 2 diabetes (13).

Among minerals, magnesium, copper, potassium, and iron are present in cocoa and chocolate in significant amounts (14). Magnesium, copper, and potassium exert a cardio protective role (15–17), while iron, mainly present in dark chocolate, contributes to the 25% of the U.S. recommended dietary allowance for middle-aged man, thus preventing anemia outcome (18).

Finally, with regard to polyphenol composition, catechins, anthocyanins, and proanthocyanidins are the most abundant class of compounds contained in cocoa powder (19). In particular, flavanols are presented as monomers, e.g., monomers (+)− and (−)− isomers of catechin and epicatechin (epi), and, in addition their derivatives are build-up of epi subunit polymers (proanthocyanidins) (19–21). Minor components are represented by phenolic acids, flavonols, and their glycoside, some stilbenes, simple phenol, and isocoumarin (22–24). Among anthocyanins, cyanidins-3-α-l-arabinoside and cyanidin-3-β-d-galactoside are the most represented compounds (18). (−)− epi accounts for the 35% of the total phenolic content, while (+)− catechin, (+)− epigallocatechin and gallocatechin are minor constituents. Procyanidins are present as dimers, trimers, and oligomers of flavan-3, 4-diols, linked by 4 → 8 or 4 → 6 bounds (20, 25, 26).

As far as bioavailability of cocoa is concerned, monomeric and polymeric flavanols are rapidly absorbed in the small intestine upon ingestion with a maximal plasma concentration after 2 h from intake (27). Elimination of flavanols is completed after 6 h from ingestion (28). However, absorption not only depends on flavanol chemistry but also on their structural isomerism and stereoisomerism (29). Also, the range of polymerization seems to determine their bioavailability (30). Once absorbed under form of monomers, flavanols are transformed into metabolites detectable in plasma and urine, such as (−)− epi as sulfate, glucuronides, or methyl conjugated forms (31, 32). On the other hand, polymers and monomers of unabsorbed flavanols undergo colonic microbiota catabolism, and valero lactones and valeric acids represent the so-called first-step microbiota-derived catabolites (33, 34). Instead, a number of phenolic acids constitute intermediate and last-step catabolites (33, 35–37). Of note, a part of unabsorbed flavanols is excreted into the feces (33, 38, 39). In this framework, it is worthwhile emphasizing that microbiota-derived metabolites of ingested polyphenols in view of their healthy effects are object of intensive investigation (40, 41). For instance, with special reference to consumers of cocoa polyphenols, a comparison between regular consumers of chocolate and low consumers has clearly shown a significant difference in terms of metabolite profiles (42).

This review will illustrate the major effects of cocoa and dark chocolate consumption in health and disease and possible cellular and molecular mechanisms of action involved also in relation to putative therapeutic implications.

## Effects of Cocoa and Dark Chocolate on the Cardiovascular System

The cardioprotective effects exerted by polyphenols have been published long ago (43, 44). Since then, a series of studies supported the protective effects of cocoa and chocolate intake on the cardiovascular system. First of all, there is robust evidence that consumption of flavanol-rich cocoa leads to beneficial effects in healthy individuals. A study has documented that vasodilation was the main effect observed as a consequence of nitric oxide (NO) release following cocoa ingestion (45). In this connection, improvement of endothelial function was higher in older (>50 years) than in younger (<50 years) healthy individuals, as assessed by flow-mediated dilation (FMD) measurement (46). In this context, *ex vivo* flavanol-induced relaxation of pre-constricted rabbit aortic rings, as well as *in vivo* increase in FMD were abrogated by inhibition of NO synthase, thus supporting the role of NO in the amelioration of endothelial function (21).

In an acute study, the effects of dark chocolate and white chocolate were evaluated in healthy participants monitoring variations of FMD and BP (47). Actually, dark chocolate was more effective than white chocolate in lowering the above mentioned parameters. In the second phase of the study, sugar-free but not sugared cocoa consumption led to a significant reduction of both systolic and diastolic BP in comparison with placebo (48). In similar trials, the effects of consumption of solid dark chocolate on endothelial function of healthy individuals were determined (49). A significant increase in FMD was observed in high-flavonoid intakers of dark chocolate (46 g) when compared to low flavonoid intakers once a day for 2 weeks. Shiina et al. (50) reported in healthy individuals an increase of coronary flow velocity reserve following consumption of 45 g of flavonoid-rich dark chocolate in comparison to flavonoid-free white chocolate. All these evidences are confirmed by studies conducted in Kuna islanders who commonly ingest higher amounts of cocoa than mainlanders (51, 52). In fact, in the former urinary flavanol metabolites were more elevated than in the latter, and this evidence correlates with low rate of cardiovascular disease (CVD), diabetes, and cancer in islanders.

On the other hand, in subjects at risk for CVD, consumption of cocoa led to results of clinical value, such as increase in nitrosylated and nitrosated species and FMD (53). Same results were obtained in smokers who consumed high flavanol cocoa beverages for 7 days (54). FMD increase was maintained on each day after a washout of 1 week. Also in diabetics, chronic consumption of cocoa three times a day for 30 days, containing 321 mg of flavanols, led to higher increase in FMD in comparison to the low-flavanol cocoa group (55). Conflicting results have been obtained in patients with coronary artery disease (CAD). For instance, in a study involving 40 CAD patients who consumed a chocolate bar and cocoa beverage, containing 444 mg of flavanols for 6 weeks, no significant differences were seen in terms of endothelial function measurement and high-sensitivity CRP, oxidized LDL, lipids, glucose, and insulin determination in comparison to placebo-treated patients (56). Conversely, in another research, 16 CAD patients were divided into 2 groups, one receiving high flavanol cocoa (375 mg) and another one consuming low flavanol cocoa (9 mg) 2 times a day for 30 days, randomly (57). More significant results were observed in the high flavanol group in comparison to the low flavanol group in terms of increase in both FMD and mobilization of circulating angiogenic cells and decrease in BP. Furthermore, other two studies have clearly demonstrated the effects of daily chocolate consumption on coronary circulation. In heart transplanted individuals, intake of 40 g of dark chocolate led to increase in coronary artery diameters and endothelium-dependent coronary vasomotion 2 h after intake of flavonoid-rich dark chocolate with a significant decrease in platelet aggregation (58). Parallely, increase in serum epi was recorded.

With regard to the mechanisms of action of NO on endothelium function, there is evidence that it causes arterial vasodilation in healthy subjects, while in individuals at risk for cardiac disease NO response is decreased while oxidative stress is increased (59–61). Furthermore, NO exerts anti-inflammatory activity *in situ* by decreasing leukocyte recruitment and platelet aggregation (62). In this framework, our own studies have clearly demonstrated that human healthy peripheral monocytes are great producers of NO when *in vitro* stimulated with red wine polyphenols (63). Then, in addition to endothelial cells, which are another source of NO, also monocytes contribute to the NO-mediated vasodilation and cardioprotection.

Taken together, these evidences clarify why polyphenols, even including those from cocoa and dark chocolate, are able to improve endothelial function in health and disease *via* NO release.

With regard to the mechanisms of NO release, all polyphenols regardless of their sources are able to activate endothelial NO synthase (eNOS), thus leading to NO generation (64). The administration of pure (−)− epi seems to reproduce the effects of cocoa-induced synthesis of NO on human coronary artery endothelial cells through eNOS activation *via* phosphatidylinositide 3-kinases/protein kinase B, also known as AKT/protein kinase A and Ca^2+^-calmodulin (CaM)/CaM K II pathway (64). Moreover, by inhibiting phospholipase C, evidence has been provided for the existence of a putative epi receptor on the cellular plasmalemma (64).

Once released, NO is able to activate the soluble guanylate cyclase in the smooth muscle cells and platelets with increase of cyclic guanosine monophosphate (cGMP) (65, 66). The subsequent inhibition of calcium flux and decrease of cytosolic calcium concentration give rise to smooth muscle cell relaxation and platelet aggregation inhibition (see also next paragraphs) (65, 66). Furthermore, cGMP is able to increase cyclic adenosine monophosphate (cAMP), which, in turn, activates prostacyclin (65–67). Quite interestingly, prostacyclin acts as a vasodilator in synergy with NO, thus contributing to protection from thrombosis. Furthermore, the anti-inflammatory and vasoprotective properties of prostacyclin are enhanced by its capacity to decrease plasma leukotrienes (68, 69).

**Figure 1:**
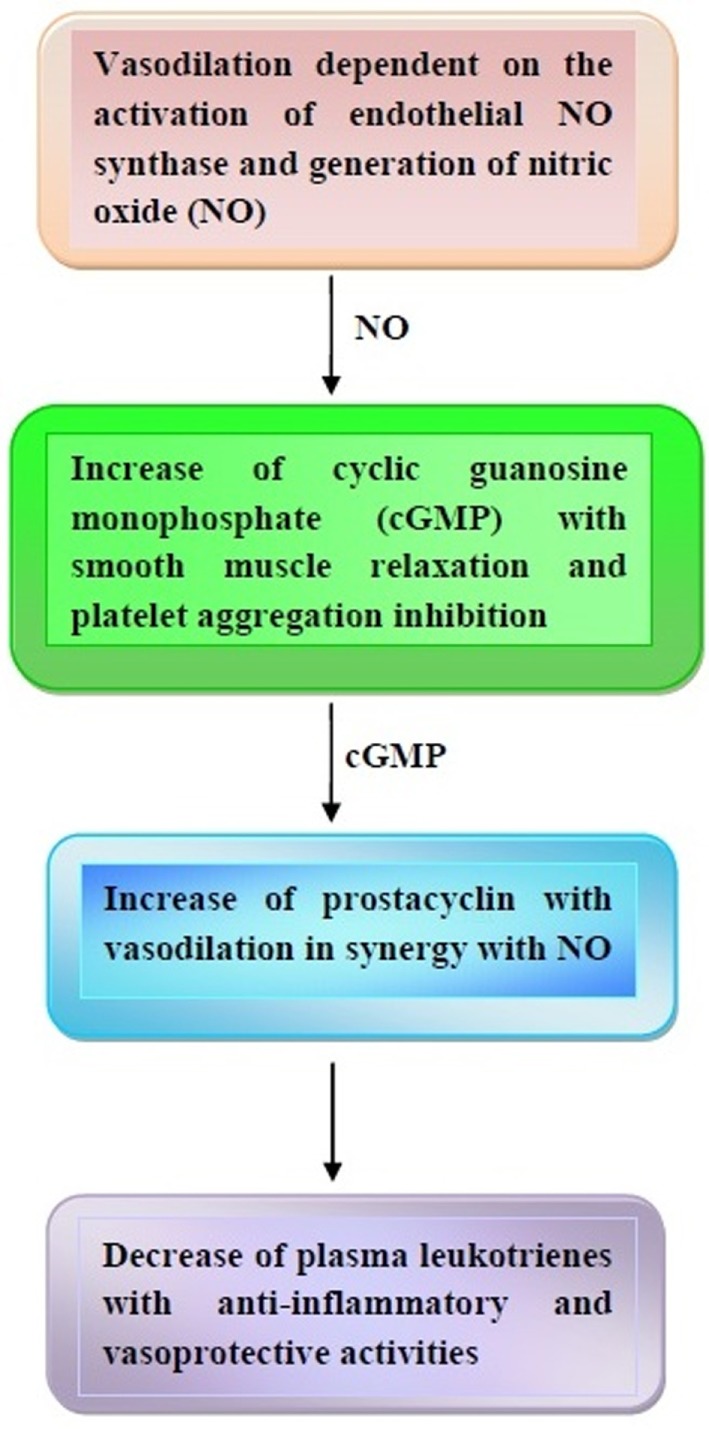
Major effects of cocoa and dark chocolate on the cardiovascular system. In response to cocoa and dark chocolate ingestion, a cascade of events takes place based on the nitric oxide (NO) and cyclic guanosine monophosphate (cGMP)-induced vasodilation and prostacyclin-mediated anti-inflammatory effects. Other details are contained in the text.

Some of the major vasoprotective effects of cocoa and dark chocolate are illustrated in Figure 1.

Finally, NADPH oxidase seems to be another target of NO activity. In fact, cocoa polyphenols reduce levels of NADPH oxidase, which generates ${\text{O}}_{2}^{-}$ that, in turn, scavenges NO. Therefore, its inhibition increases levels of NO (70, 71).

Another important target of polyphenol-rich cocoa is represented by platelets. First of all, platelets can *per se* release NO under influence of flavanols (72), thus contributing to vasodilation. Cocoa-mediated inhibition of platelet aggregation has been shown to depend on the decrease of thromboxan (TX) A2 synthesis and antagonism at TXA2 receptors (73–75). Furthermore, other possible mechanisms of action are represented by the inhibition of platelet–leukocyte interaction since cocoa flavanols are able to inhibit CD62P expression on activated platelets (76–78). Of note, CD62P binds P-selectin glycoprotein ligand-1 on leukocytes, thus mediating the platelet–leukocyte interaction.

A series of studies have demonstrated the cocoa’s platelet inhibitory effects in healthy individuals and in heart transplant patients (79–81) who had consumed cocoa or dark chocolate. Taking into account that platelet activation greatly contributes to the inflammation and thrombosis in the progression of CVD, their inhibition by polyphenol-rich diets, even including consumption of cocoa and dark chocolate, is of clinical relevance.

The cocoa-mediated decrease of BP can be ascribed to several mechanisms. Increase in NO may explain the anti-hypertensive effects of cocoa (82). In addition, there is also evidence that flavanols and flavonol are able to *in vitro* inhibit angiotensin-converting enzyme (ACE) activity (83, 84). ACE, in turn, acts on the renin–angiotensin system, cleaving angiotensin I into angiotensin II with release of vasopressin or aldosterone and anti-diuretic hormone and increase in sodium and water retention. ACE also inhibits bradykinin and kallidin, which act as vasodilators (85).

In terms of effects of cocoa on serum lipid profile, a number of studies have clearly demonstrated that consumption of cocoa leads to increase in HDL while lowering LDL (86, 87). The same holds true also in the case of ingestion of high-polyphenol chocolate (88). Basically, same results were reported in individuals fed cocoa beverages containing only cocoa powder. Furthermore, a meta-analysis study confirmed the ability of cocoa to reduce LDL cholesterol and total cholesterol in subjects at high cardiovascular risk (89, 90). Also, inhibition of LDL oxidation is another effect of both cocoa and dark chocolate consumption (89–92). Conversely, other studies failed to demonstrate significant differences in serum lipids between consumers of high-flavonoid chocolate and consumers of low-flavonoid chocolate (49, 93). Similarly, in other three studies, no effects of cocoa beverages on serum lipids were observed (94, 95).

## Cocoa and Dark Chocolate Effects on the Central Nervous System (CNS) and Behavior

The beneficial effects of polyphenols on the CNS have extensively been described in human and animal studies. The majority of research has been conducted with polyphenols derived from soy, berries, wine, tea, and curcuma and much less from cocoa and chocolate (96). Also, flavonoids extracted from *Ginkgo biloba* have been reported to retard memory loss, dementia, and Alzheimer’s disease (AD) progression. However, data are still controversial (97, 98). In a series of researches, the anti-inflammatory activity exerted by polyphenols on the CNS has been documented. Curcumin extracted from *Curcuma longa* root was able to reduce the production of tumor necrosis factor (TNF)-α, interleukin (IL)-6, and reactive oxygen species (ROS) from primary astrocytes *in vitro* stimulated with 1-methyl-4-phenylpiridinium ion (MPP+) (99). Moreover, curcumin increased levels of IL-10 and glutathione. Curcumin also decreased levels of toll-like receptor (TLR)-4, as well as of NF-κB, interferon regulatory factor 3, MyD88, and TIR-domain-containing adapter-inducing interferon-β otherwise enhanced by MPP+ (100). Similarly, epi and resveratrol have been found to exert neuroprotective activity modulating TLR-4/NF-κB/signal transducer and activator of transcription (STAT) signaling pathways (100).

**Figure 2:**
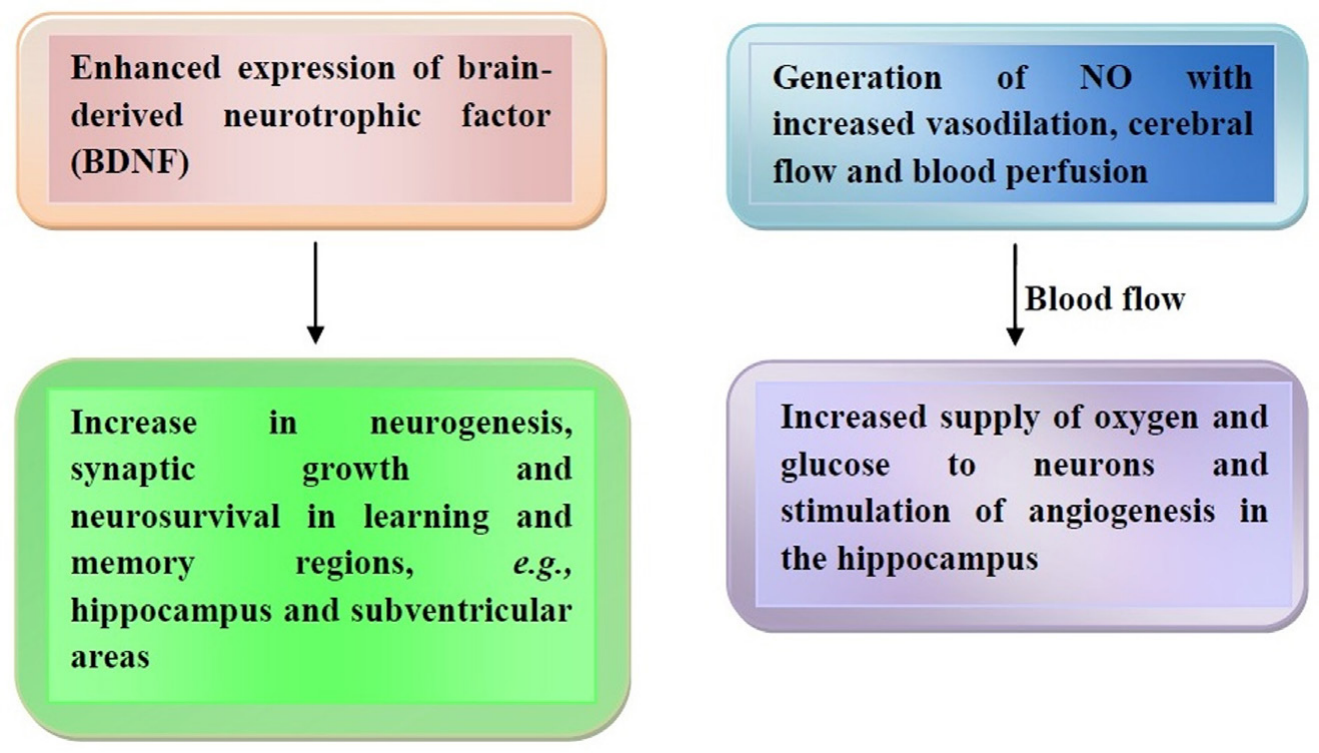
Cocoa flavanol-mediated brain effects. Release of brain-derived neurotrophic factor (BDNF) with increased neurogenesis and neurosurvival (left panel) and nitric oxide (NO)-mediated increase of cerebral blood flow (right panel) are the major effects exerted by cocoa flavanols. Further details are illustrated in the text.

Others have reported that polyphenols can interact with some signaling pathways, such as mitogen-activated protein and phosphoinositide-3-kinase (PI3-kinase)/AKT, thus leading to gene expression and protein synthesis for long-term potentiation and long-term memory occurrence (101). Flavonoids modulate transcription factors *via* protein kinase inhibition (102), while inducing the expression of brain-derived neurotrophic factor (BDNF). This factor contributes to neurogenesis, synaptic growth, and neuron survival in certain learning and memory brain regions, such as the hippocampus and subventricular areas (103, 104). Another mechanism is based on the generation of NO that leads to vasodilation and increased cerebral blood flow and blood perfusion in the context of the CNS as well as of the peripheral nervous system (105, 106). Such an increased blood flow is able to supply oxygen and glucose to neurons, also getting rid of waste metabolites in the brain and sensory organs (107, 108) while stimulating angiogenesis in the hippocampus (109). The effects of cocoa flavanols on the brain are represented in Figure 2.

Different cocoa flavonoid effects on Parkinson’s disease (PD) have been reported. In PD, death of neurons in *substantia nigra* depends on the generation of 5-S-cysteinil-dihydrobenzothiazine ROS mediated-effects (110). Quite interestingly, neuronal damage mediated by 5-S-cys-DA is dramatically mitigated by quercetin, hesperetin, and caffeic acid, which are derivatives of catechin and epi (110). Neuroinflammation is another hallmark of PD pathogenesis (111). Microglia response plays the major role in the progression of neuronal degeneration and, consumption of cocoa flavonoids, e.g., quercetin, leads to anti-inflammatory effects (112). In particular, quercetin behaves as certain kinase inhibitors that exert anti-inflammatory effects on glial cells (112), likely preventing excitotoxic death in neurons (113). In relevance to the above cited anti-inflammatory effects, evidence has been provided that fermented grape marc (FGM) polyphenols have the capacity to reduce *in vitro* release of granzyme B from healthy peripheral human cytotoxic T cells, thus lowering their neurotoxic potential (114). By analogy, cocoa polyphenols may exert similar neuroprotective activity.

Alzheimer’s disease is characterized by an increased production of amyloid (A)β oligomers, which activate microglia with release of inflammatory mediators and neuronal death (115). In an *in vitro* model of human AD, cocoa polyphenolic extracts have been shown to exert not only antioxidant effects but also to afford neuroprotection (116). This last effect has been attributed to the activation of BDNF survival pathway either on Aβ plaque-treated cells or on Aβ oligomer-treated cells, thus, ultimately, leading to reduction of neurite dystrophy. Resveratrol, a non-flavonoid component of polyphenols (117), exhibited neuroprotective effects in AD. In fact, it promoted non-amyloidogenic breakdown of the amyloid precursor proteins and removal of neurotoxic Aβ peptides. It is likely that also cocoa polyphenols may exhibit similar activities. Another protective mechanism mediated by cocoa polyphenols is the activation of NAD(+)-dependent histone deacetylase enzymes, termed Sirt (118). In particular, in the course of AD, reduced levels of Sirt1 upregulate NF-κB, which, in turn, trigger inflammation and enhances Aβ toxicity (119, 120).

Another experimental study based on the administration of dark chocolate to a non-transgenic AD obese model showed a reduction of hyperglycemia and cholinesterase activity in the hippocampal tissue homogenates and improvement of the cognitive performance (121).

Another neurotrophic effect of cocoa flavonoids is represented by their ability to increase cerebral blood flow in healthy young subjects, as assessed by functional magnetic resonance imaging (FMRI) (122). This effect was observed 3 h after cocoa consumption. Furthermore, such an increased blood flow to gray matter has been shown to account for angiogenesis as well as growth of new hippocampal neurons involved in the memory processing (110). In this context, evidence has been provided that increase in blood flow in the middle cerebral artery may account for protective effects in the course of dementia and stroke (123).

The effects of cocoa flavonols on PD and AD progression are represented in Table 1.

**Table 1:** Beneficial effects of cocoa flavanols on the progression of Parkinson’s disease (PD) and Alzheimer’s disease (AD).

PD	AD
Inhibition of 5-*S*-cysteinil-dihydrobenzothiazine-mediated neuronal damage (110)	Activation of brain-derived neurotrophic factor on amyloid (A)β plaque-treated cells or on Aβ oligomer-treated cells (116)
Anti-inflammatory effect mediated by quercetin on glial cells, behaving as certain kinase inhibitors, thus preventing excitotoxic death in neurons (112, 113)	Activation of NAD(+)-dependent histone deacetylase enzymes known as sirtuins (118)
	Reduction of hyperglycemia and cholinesterase activity in the hippocampus with improvement of cognitive functions (121)

With special reference to the influence on behavior, a series of studies have demonstrated that palatable chocolate consumption is able to improve mood in a more significant manner than that performed by a non-palatable chocolate (124, 125). Palatability seems to be related to the chocolate-mediated release of opioids, such as β-endorphins in the hypothalamus (126), thus producing an analgesic effect (127).

Also, cognitive function has been shown to be improved by cocoa beverages with reduction of mental fatigue (128). However, others did not find any significant change of cognitive tests in comparison to placebo group in healthy old subjects who consumed cocoa-enriched beverages and dark chocolate (129).

Chocolate consumption seems to stimulate different brain areas, especially chemosensory areas, such as insula, prefrontal region, caudomedial and caudolateral orbitofrontal cortex (130). According to FMIR, a significant taste-related activation in the orbitofrontal and insular cortices was reported (131). Also, chocolate color modulates brain activity with significant reduction in theta activity. This implies reduced levels of attention and higher levels of distraction (132). Finally, the sight of chocolate generated more activation in chocolate cravers than non-cravers in the medial orbitofrontal cortex and ventral striatum (133).

## Effects of Cocoa and Dark Chocolate on Intestinal Inflammation

Over the past years, plant-derived polyphenols have been experimented in *in vitro* and *in vivo* models of intestinal inflammation in view of their anti-inflammatory potential (134, 135). Interesting results have been obtained *in vitro* treating Caco-2 cells with cocoa polyphenols (134). Such a treatment led to induction of prostaglandin E2 synthesis *via* cyclooxygenase (COX)-1 effect, which may be involved in the maintenance of mucosal integrity. On the other hand, the murine model of dextran sulfate sodium (DSS)-induced colitis has been used for investigating the effects of polyphenol administration. For instance, administration of cocoa FGM-derived polyphenols to DSS-induced colitis mice led to a partial but significant abrogation of intestinal length reduction, while levels of TNF-α and IL-1β significantly dropped in inflamed colon homogenates in comparison to untreated colitis animals (136). Similar results have been documented by Pérez-Berezo and associates (137) in rats with DSS-induced colitis administered with a cocoa-enriched diet. Decrease of colonic cellular infiltrates was paralleled by reduction of serum TNF-α and colon inducible (iNOS) activity. However, despite the reported changes, no clinical improvement was recorded in rats. In a murine model of DSS-induced colitis, Andújar and associates (138) reported that administration of cocoa polyphenols mitigated symptomatology accompanied by reduction of neutrophil infiltration, NO generation, expression of COX-2 and STAT-1 and STAT-3 (138) as well reduction of IL-1β, IL-6, and TNF-α from peritoneal macrophages (138). These modifications of biomarkers were associated to improvement of colitis. However, no inhibitory effect of NF-κB was detected in the nuclear extract of colon. Conversely, cocoa consumption by healthy volunteers led to a significant reduction of NF-κB in peripheral blood mononuclear cells (PBMCs), thus suggesting an inhibitory effect on the release of pro-inflammatory cytokines (139).

In the light of these results, addition of polyphenols to enteral nutrition in patients with inflammatory bowel disease may be beneficial in view of their ability to induce phase II antioxidant and detoxifying proteins, thus preventing or improving the inflammatory status (140).

## Effect of Cocoa and Dark Chocolate on Obesity

Evidence has been provided that cocoa administration to rats decreased visceral adipose tissue, thus changing the expression of genes, which are involved in the generation of enzymes and molecules for the occurrence of FA synthesis and thermogenesis in liver and white adipose tissue (141). In a study conducted in 12 females, dark chocolate smelling was assessed for evaluating appetite response (142). This led to a satiation response, which inversely correlated with ghrelin levels. Since ghrelin is involved in adiposity induction (143), one can conclude that chocolate may reduce appetite, preventing weight gain. Furthermore, evidence has been provided that flavonoids act on peroxisome proliferator-activated receptors (PPARs), thus behaving as agonists of PPAR-α and partial agonist of liver X receptor α (144–146). In addition, increased expression of PPAR-γ, which, in turn, increases expression of adiponectin and glucose transporter 4, is another mechanism elicited by cocoa flavonoid consumption (147). These events may lead to reduced lipogenesis, induction of lipolysis, and increase in adiponectin secretion. Adiponectin also reduces lipid deposition and insulin resistance, thus mitigating obesity.

**Figure 3:**
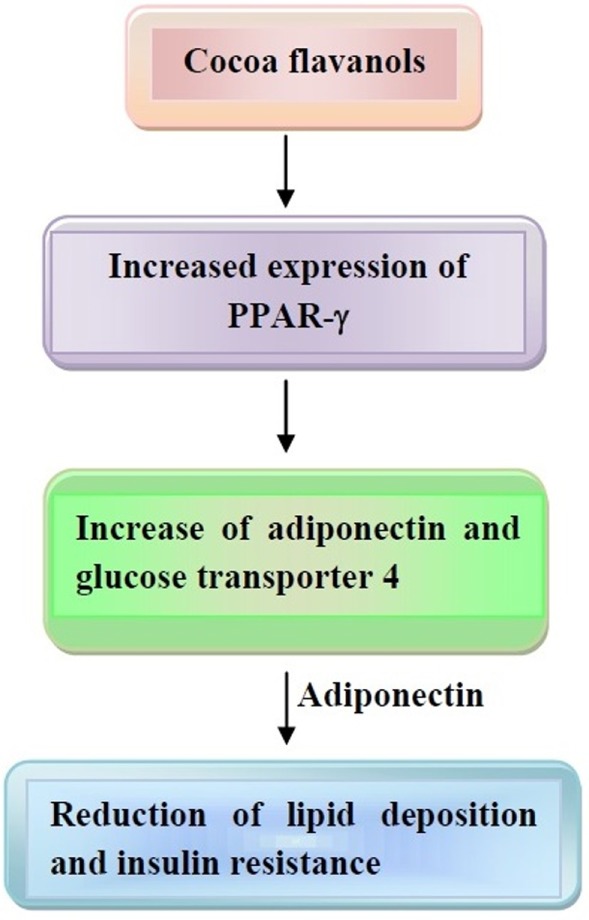
Mechanisms of action of cocoa flavanols on obesity development. Increased expression of peroxisome proliferator-activated receptor (PPAR)-γ and adiponectin leads to reduction of lipid deposition and insulin resistance. Other details are present in the text.

These last mechanisms are depicted in Figure 3.

Another important function of cocoa flavanols related to obesity is the delay of LDL oxidation. For example, they decrease F2-isoprostane levels, which represent *in vivo* markers of lipid peroxidation (148, 149). As result of LDL oxidation inhibition, decrease in atherosclerotic lesions in hypercolesterolemic rabbits treated with a diet enriched in cocoa powder for 24 weeks has been documented (150). Conversely, other researchers failed to confirm inhibition of LDL oxidation in rats treated with cocoa polyphenols for 2 weeks (151). In healthy human volunteers, evidence has been provided that cocoa consumption led to decrease of F2-isoprostane and thiobarbituric acid reactive substances, which are biomarkers of LDL oxidation and lipid peroxidation, respectively (152–154). Quite interestingly, in healthy humans, cocoa consumption increased plasma HDL cholesterol (92, 155), while decreasing plasma triglycerides (156–158). These results suggest the healthy benefits of cocoa consumption by changing the expression of genes involved in FA catabolism.

## Effect of Cocoa and Dark Chocolate on the Immune System

Several studies of our group have been conducted on the effects of red wine or FGM-derived polyphenols on the immune cells. In murine models of asthma, FGM-derived polyphenols were able to mitigate symptomatology (159) when orally administered. In human studies, both red wine and FGM-derived polyphenols were able to induce *in vitro* activation of T regulatory (Treg) cells and release of IL-10, which, in turn, mediates anti-inflammatory activity (160, 161). FGM-derived polyphenols were also able to reduce the respiratory burst of healthy neutrophils and monocytes and abrogate basophil as well as rat mast cell degranulation *in vitro* (162, 163).

With special reference to cocoa flavanols, their *in vivo* administration to experimental animals has clearly demonstrated changes in the lymphoid organs. In rats, a diet based on 10% cocoa led to thymocyte differentiation and upregulation of thymic antioxidant defenses (164). Same dietary regimen increased splenic B cell percentage and decreased splenic T helper (h) cell frequency in rats (165, 166). In the gut of rats, changes in lymphomonocyte profile and Th cells frequency at Peyer’s patches and mesenteric lymph node levels were noted following cocoa administration (165, 166).

The *in vitro* effects of cocoa on cytokine secretion are quite controversial. Increase in TNF-α, IL-1β, IL-6, and IL-10 from human PBMCs stimulated with flavanol fractions of cocoa have been reported (167). Conversely, following cocoa stimulation reduced production of TNF-α, monocyte chemoattractant protein-1, and NO by endotoxin-stimulated macrophages has been documented (168). In the same set of experiments, it was reported that cocoa polyphenols were able to modulate endotoxin activation of granulocytes (168). With special reference to Th cells, a cocoa diet in rats increased IL-4 production (a Th2 cytokine) from splenocytes (169). Secretion of interferon-γ from rat splenic Th1 cells was unmodified (166, 170), increased (171), or *in vitro* suppressed by cocoa extracts (172). Of note, cocoa diet did not modify rat IL-10 production (166, 173).

A series of experiments with procyanidin C1 using RAW 264.7 macrophages have clarified some important aspects of cocoa-mediated immunomodulation. In this respect, procyanidin C1 significantly enhanced levels of iNOS-mediated NO generation by activated macrophages (174). In addition, it increased the expression of the costimulatory molecules CD80 and CD86, thus potentiating antigen presentation to T cells (175). With regard to signaling pathways, procyanidin C1 was able to trigger phosphorylation of MAPKs, even including p38 and extracellular signal-regulated kinase as well as of nuclear factor of kappa light polypeptide gene enhancer in B-cells inhibitor-α with subsequent activation of NF-κB. These findings were confirmed by using specific inhibitors of NF-κB and MAPK, which hampered pro-inflammatory cytokine production in the same experimental model.

Transforming growth factor (TGF)-β1 is a pleiotropic cytokine involved in tissue repair and regeneration (176, 177). Therefore, the effects of cocoa flavanols on the production of this cytokine were also evaluated in human subjects (178). Results pointed out that in healthy subjects cocoa consumption was able to regulate TGF-β1 production with an increase in low producers and a decrease in high producers (178). Of note, low levels of TGF-β1 were detected in patients with advanced atherosclerosis (178), while its excessive production has been shown to lead to cardiac fibrosis (179). Therefore, cocoa consumption by individuals with cardiovascular risk leads to modulation of TGF-β1 production, thus leading to protective functions.

Cocoa flavanols have been shown to regulate secretion of IL-5. Smaller molecular weight flavanol fractions were able to *in vitro* enhance IL-5 release by healthy human PBMCs, while larger molecular weight flavanol fraction decreased its release (180). The cocoa-induced increase of IL-5 may be indicative of a switch of the humoral immune response toward secretory IgA production, thus reducing the risk for caries and periodontal disease (180).

Finally, the effects of cocoa polyphenols on the composition of intestinal microbiota need to be mentioned. According to studies of Tzounis and associates (181, 182), Spencer and associates (183), and Massot-Cladera and associates (184), flavanol monomers and dimers are absorbed in the small intestine, while procyanidins are metabolized in the colon by the intestinal microbiota into a variety of phenolic acids, which are also absorbed. All absorbed products are metabolized in the liver and eliminated in the urine, and, partly, in the feces. In a human trial conducted on healthy volunteers, consumption of a high-cocoa flavanol beverage for 4 weeks, containing 494 mg flavanols, significantly increased the growth of *Lactobacillus* spp. and *Bifidobacterium* spp. in comparison to a low cocoa flavanol drink (182). Usually, these bacteria are able to maintain an anti-inflammatory status in the bowel with activation of Treg cells and production of IL-10 (185), thus suggesting that cocoa polyphenols may behave as prebiotics and trigger a tolerogenic pathway in the gut.

**Figure 4:**
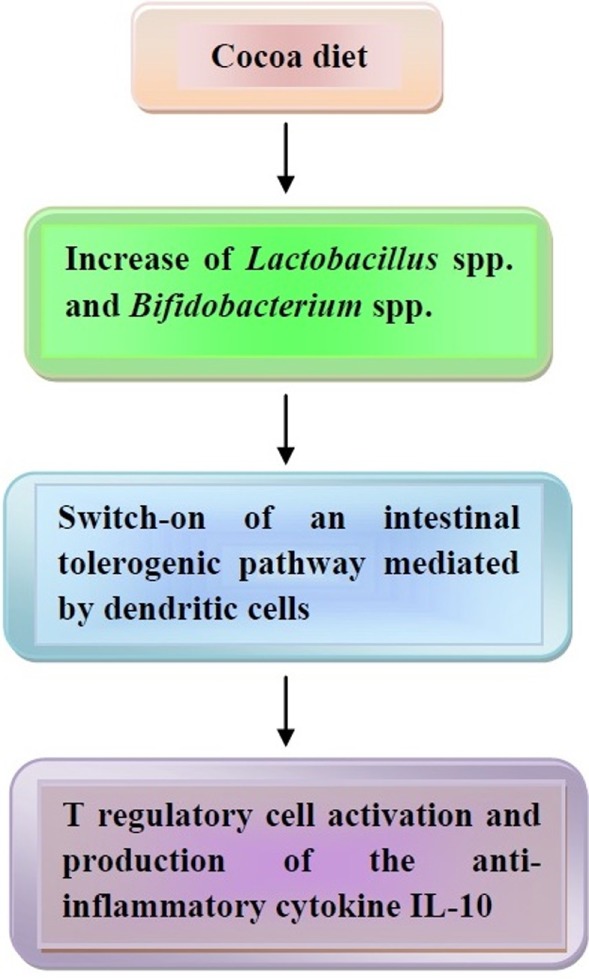
The effects of coca-enriched diet on human microbiota. Cocoa diet modifies the intestinal microbiota, thus leading to a tolerogenic pathway with release of the anti-inflammatory cytokine interleukin (IL)-10. In the text, further details are illustrated.

The effects of cocoa on microbiota are illustrated in Figure 4.

At the end of this section, one should mention the effects of (−)− epi, (+)− catechin, and dimeric flavonols on NF-κB, a transcription factor involved in immune cell activation.

The abovementioned compounds are able to inhibit NF-κB activation, and, in particular the phorbol mirystate acetate (PMA) DNA binding activity, thus resulting in IL-2 production decrease (185). Inhibition of binding activity is provoked by a blockade of the binding of active NF-κB to the DNA KB motifs. Finally, pretreatment with flavanols leads to decrease of PMA-stimulated intracellular oxidants, which is an early event in NF-κB triggering.

## Conclusion

There is wealth of evidences concerning the relationship between health status and integrity of vascular and neurological functions. As extensively described in the previous sections of this review, cocoa and dark chocolate-mediated induction of NO leads to vasodilation as well as inhibition of COX-2, CRP, and atherogenesis (186, 187). In addition, NO acts in concert with BDNF in order to modulate neural progenitor cell growth and synaptic metabolism for appropriateness of cognitive functions (188–190). Quite interestingly, release of NO at the thalamus level contributes to the adequate functioning of the neurovascular unit *via* increased blood flow and volume in the context of the brain (191, 192). Furthermore, polyphenols, even including those from cocoa, exert antioxidant effects, thus increasing neurological functions also preventing age-dependent damage (193). In synthesis, by analogy to other plant-derived polyphenols, cocoa flavanols may exert beneficial effects *via* activation of eNOS, inhibition of the NADPH oxidase and ROS production, downregulation of NF-κB, and regulation of MAPK and cAMP response element-binding protein pathways (194–197). In aging, especially neurological functions become deteriorated, and NO and aging seem to be interconnected. For instance, alterations of NOS have been detected in aging brain, thus influencing memory (96, 198, 199).

Conclusively, in the light of the above considerations, cocoa and dark chocolate-based diet may be beneficial in aged people for improvement of the neuro–cardiovascular connectivity.

## Author Contributions

All authors equally contributed to the compilation of the present review.

## Conflict of Interest Statement

The authors declare that the research was conducted in the absence of any commercial or financial relationships that could be construed as a potential conflict of interest.
